# Editorial: Tumor-draining lymph nodes

**DOI:** 10.3389/fimmu.2024.1533289

**Published:** 2024-12-16

**Authors:** Maximilian Seidl, Nikolas Hendrik Stoecklein, Dennis Jones

**Affiliations:** ^1^ Institute of Pathology, Heinrich-Heine University and University Hospital of Düsseldorf, Düsseldorf, Germany; ^2^ Clinic for General, Visceral and Pediatric Surgery, University Hospital Düsseldorf, Düsseldorf, Germany; ^3^ Department of Pathology & Laboratory Medicine, Chobanian & Avedisian School of Medicine, Boston University, Boston, MA, United States

**Keywords:** prognosis, immune suppression, metastasis, tumor draining lymph node, tumor immunology, immunotherapy

Lymph nodes are essential for developing tumor-specific effector responses, as they comprise all the necessary cell types organized in specific microanatomical compartments to effectively initiate adaptive immune responses. However, lymph nodes that drain the tumor are usually the first metastatic site in most solid cancers, reflecting their functional suppression as the disease progresses. The articles in this Research Topic “*Tumor-draining lymph nodes*” provide insights into the connection between the functional status of tumor-draining lymph nodes (TDLNs) and patient survival, as well as their potential to enhance responses to immunotherapy.

As reviewed by Lei et al., TDLNs offer important prognostic information, as the histological detection of metastatic cancer cells within them yields insights into disease progression. Historically, lymph node metastases were viewed as indicative of an aggressive tumor. More recently, however, studies have shown that cancer cells can exit lymph nodes and directly colonize distant organs (1, 2). The study by Kooreman et al. suggests that the shape of TDLNs may be prognostic across different breast cancer subtypes, independent of cancer cell presence. Lymph nodes are bean-shaped organs that exhibit remarkable plasticity, undergoing architectural changes in response to factors such as aging, immune cell expansion, and tumor growth. Morphometric measurements made by Kooreman et al., particularly the ratio of the long to short axes of cancer-free TDLNs, were found to correlate with the presence of tumor-infiltrating lymphocytes (TILs) in the primary tumor and overall survival outcomes. Compelling data demonstrate that elongated nodes are predictive of fewer TILs, as well as poor overall survival and disease-free survival. In contrast, nodes with a more rounded shape may have better immune function, as they are associated with a higher presence of TILs. The study also adds to growing evidence that normal tissue in lymph nodes is progressively replaced by fat as individuals age, which may indicate a decline in nodal function (3). It would be valuable to explore whether magnetic resonance imaging used for breast cancer diagnosis could also predict patient prognosis and therapeutic response, based on lymph node adiposity and shape.

Circulating naïve lymphocytes in the blood enter lymph nodes through high endothelial venules (HEV). In the paracortex, T cells engage with dendritic cells and are presented with cognate antigen for priming. HEVs in metastatic lymph nodes have previously shown to be impaired by secondary tumors (4). The study by Bravo et al. examined metastatic lymph nodes and in some cases identified focal accumulations of dendritic cells and naive lymphocytes next to HEVs–a structure they termed Favoring Antigen-Presenting Structures (FAPS). These structures were associated with improved distant-metastasis free survival in melanoma patients receiving vaccination with tumor antigens. Notably, patients were vaccinated after the resection of metastatic lymph nodes. This suggests that metastatic nodes may retain residual function before resection and potentially influence primary tumor burden, as patients with abundant FAPs exhibited a low primary tumor burden. This finding also suggests that vaccination may be more effective in patients with minimal residual disease compared to those with advanced disease. Further, when examined from an immunological perspective, beyond merely assessing the presence or absence of metastases, such analysis could potentially inform responses to vaccination or immune checkpoint therapy. Yet, the observed vaccine responses following the removal of TDLNs align with recent findings suggesting that distant lymph nodes may compensate for the loss of TDLNs (5).

Although the use of antitumoral immune therapies, such as PD-L1/PD-1 and CTLA4 checkpoint inhibition is becoming more common, it is still difficult to identify patients who will benefit from existing therapies, even within the same tumor type. Gaining a better understanding of the tumor-derived immunomodulation that occurs in draining lymph nodes is crucial to the development of effective immunotherapy strategies. Current therapeutic concepts are primarily based on the endpoint response of effector cells in tumors, while the critical role that TDLNs have on shaping this response has largely been neglected. The study by Piersiala et al. highlights the prognostic value of checkpoint pathways and immune cell subsets within TDLNs, shedding light on their role in shaping outcomes in oral squamous cell carcinoma. Multivariate analysis revealed that elevated levels of FoxP3+CD4+ T regulatory cells (Tregs) and TIGIT+CD8+ T cells were strongly associated with worse disease-free survival. These findings reflect distinct but synergistic mechanisms of immune suppression: Tregs actively inhibit effector immune responses, while TIGIT+CD8+ T cells represent an exhausted cytotoxic subset with reduced anti-tumor activity. Moreover, increased expression of immune checkpoints, such as PD-1 and CTLA-4 on T cells within TDLNs was significantly associated with recurrence, highlighting a profoundly immunosuppressive microenvironment that promotes immune evasion. By characterizing these immune dynamics, Piersiala et al. underscore the potential of targeting TDLNs to reinvigorate anti-tumor immunity and enhance immunotherapeutic outcomes. This work strengthens the case for TDLNs as pivotal players in tumor progression and immune modulation.

**Figure 1:**
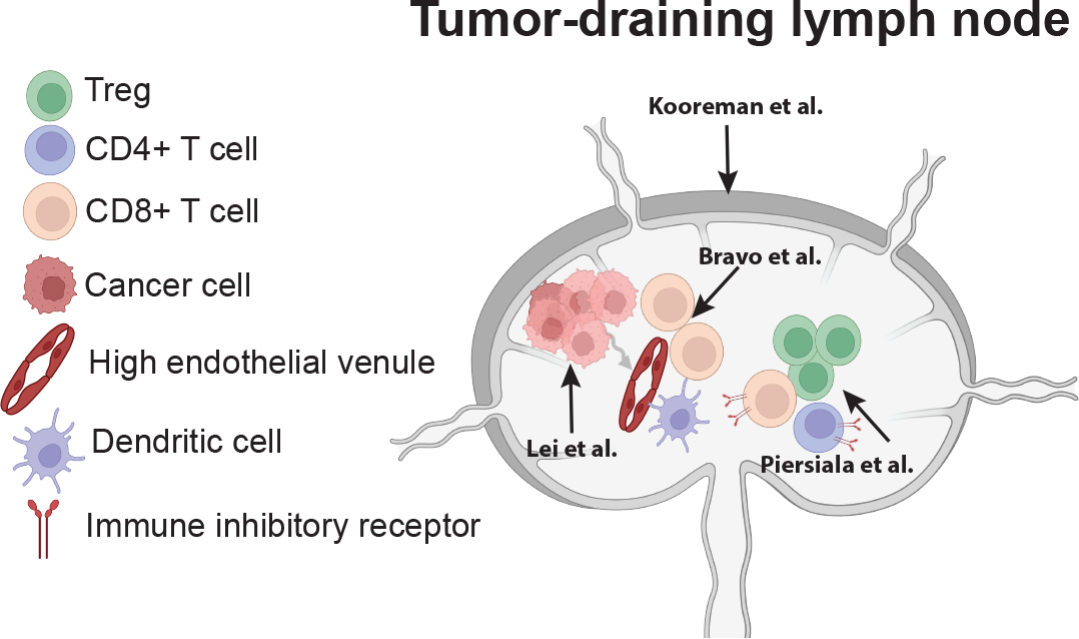
Summary of Research Topic (adapted from Lei et al.). The comprehensive review by Lei et al. summarizes cancer cell metastasis to lymph nodes and the metabolic changes cancer cells undergo to establish lymph node metastasis and further metastasize. In addition, the review highlights the stromal and cellular consequences of lymph node metastasis that define lymph node function. The study by Kooreman et al. evaluates lymph node (metastasis-free) shape and the association with nodal function and patient prognosis. Bravo et al. identify a triad of factors in the paracortex of metastatic lymph nodes—high endothelial venules, dendritic cells, and lymphocytes—that were predictive of patient response to subsequent vaccination and overall survival. Piersiala et al. identified the expression of multiple immune inhibitory receptors on T cells within tumor-draining lymph nodes as being associated with worse disease-free survival.

Overall, the findings in this Research Topic provide supportive evidence for incorporating TDLN analysis into cancer management, paving the way for innovative strategies that disrupt immune suppression and improve patient survival. **Figure 1** graphically summarizes this Research Topic.
